# The Role of Indirect Immunofluorescence (IIF) and Line Immunoassay (LIA) in the Diagnosis of Autoimmune Diseases and Their Clinical Correlation: An Observational Study From a Tertiary Care Center in Bihar

**DOI:** 10.7759/cureus.47702

**Published:** 2023-10-26

**Authors:** Avinash Kumar, Akash Bansal, Mala Mahto, Bandana Kumari, Sushil Kumar, Ayan Banerjee, Visesh Kumar, Javin B Gogoi

**Affiliations:** 1 Biochemistry, All India Institute of Medical Sciences, Patna, Patna, IND; 2 Biochemistry, All India Institute of Medical Sciences, Gorakhpur, Gorakhpur, IND; 3 Biochemistry, Mahamaya Rajkiya Allopathic Medical College (MRAMC), Ambedkar Nagar, IND; 4 Biochemistry, Soban Singh Jeena (SSJ) Government Institute of Medical Sciences and Research, Almora, IND

**Keywords:** systemic lupus erythromatosus, homogenous ana pattern, speckled ana pattern, mctd, anti-nuclear antibody (ana), line immunoassay, indirect immunofluorescence, autoimmune diseases

## Abstract

Background and aim

The presence of distinct sets of autoantigens and autoantibodies bestow these autoimmune diseases (ADs) with specific immune profiles or fingerprints, which has cleared the diagnostic dilemma associated with these ADs. This study was planned to collate and compare the reporting of indirect immunofluorescence (IIF) with line immunoassay (LIA) and their clinical correlations. This study was conducted to investigate the association between the reporting of anti-nuclear antibody (ANA) screening by IIF and ANA profile reporting by LIA. Additionally, it aimed to explore the association of ANA pattern detection by IIF with the detection of autoantibodies against nuclear antigens by LIA and the association of autoantibody detection by LIA with clinical diagnosis.

Methodology

A total of 98 samples from patients suspected of having ADs were subjected to both IIF and LIA, and results were correlated with clinical diagnosis.

Results

In the homogenous pattern noted by IIF, the clustered antigens identified by LIA included dsDNA, Nucleosome, Histone, and Mi-2. In the speckled pattern, the identified antigens were SS-A/Ro52, P0, SS-A/Ro60, SS-B/La, and U1-snRNP. On the other hand, the nucleolar pattern revealed antigens AMA M2, PCNA, and CENP-B. The centromere pattern was mostly associated with CENP-B. The speckled pattern was found to be most commonly associated with systemic lupus erythematosus (SLE). The most common autoantibody found in total ANA profile-positive samples was anti-U1-snRNP followed by anti-SS-A/Ro60 and anti-SS-B/La, and all three were found to be associated with SLE.

Conclusions

SLE was the most common AD identified in our study samples, with the speckled pattern being the most common pattern on IIF and anti-U1-snRNP being the most common ANA identified by LIA. The fluorescence pattern of IIF predicts the presence of specific antibodies. LIA should be reserved for IIF-positive but dubious cases and whose signs and symptoms are nebulous and do not match the disease dictated by IIF.

## Introduction

Autoimmune diseases (ADs) occur when the body’s natural defense system fails to differentiate between its own cells and foreign cells, thus causing the immune system to misfire and damage normal cells [1]. More than 100 ADs are known, with the common ones being systemic lupus erythematosus (SLE), mixed connective tissue disease (MCTD), Sjogren’s syndrome, etc. The major culprit behind this is the presence of self-reactive B cells (autoantibody) and or T cells directed against a person’s autoantigens, which are typically proteins or proteins complexed with the nucleic acid. These autoantibodies serve as excellent biomarkers for ADs [2]. ADs have vague and overlapping symptoms, making the diagnosis challenging [3].

The unique antinuclear antibody (ANA) screen assay uses indirect immunofluorescence (IIF) with human laryngeal epidermoid carcinoma cell line type 2 (HEp-2) cells as a substrate and is the gold standard approach for ANA detection. IIF is reported positive or negative depending on the fluorescence produced but has a limitation as it does not allow precise identification of specific antibodies. A positive IIF result indicates an interaction between serum antibodies and up to 150 different nuclear antigens of HEp-2 cells. However, only a minority of these antigens are well-described and have established associations with the disease. Reporting is also very subjective, and similar or combination of patterns may be noted for more than one antibody [4].

For quick autoantibody profiling, the human line immunoassays (LIAs) were created as a confirmatory test. LIAs are simple to conduct and have demonstrated great sensitivity and specificity [5-7]. LIAs can detect numerous autoantibodies simultaneously utilizing strips coated with different antigens, and recent developments in automation make them an enticing option for a high-throughput laboratory platform [8]. LIA is used as a confirmatory test. IIF is the gold standard test for screening ANA, but a similar pattern can be obtained for more than one antibody. LIA being very specific is very costly indeed.

This study was designed to investigate the association between reporting by IIF and ANA profile reporting by LIA. Additionally, it aimed to explore the association between ANA pattern detection by IIF and the detection of autoantibodies against nuclear antigens by LIA. Finally, the study sought to examine the association between autoantibody detection by both IIF and LIA and clinical diagnosis.

## Materials and methods

This was a cross-sectional study conducted in the Department of Biochemistry at the Tertiary Care Center of Bihar, India, from September 2019 to August 2020. All samples sent to the Biochemistry Central Laboratory from their respective departments for ANA screening were included in the study. These samples were subjected to both IIF and LIA. A total of 98 patients were enrolled in the study. This study was conducted as a part of the postgraduate dissertation. This study was approved by the Institutional Research Committee and the Institutional Ethical Committee.

ANA screening by IIF

AESKUSLIDES kit (Aesku Diagnostics, Wendelsheim, Germany) was used for ANA IIF, and the test was performed as the per manufacturer’s protocol. Results of ANA IIF were interpreted as positive control where homogenous patterns having intensity graded between + and ++++ were obtained, and negative control when it remained unstained. Each slide of ANA IIF has 12 wells coated with the HEp-2 cell line. It possesses over 100 antigens in the nucleus and cytoplasm that may have relevance to patients with AD.

**Figure 1:**
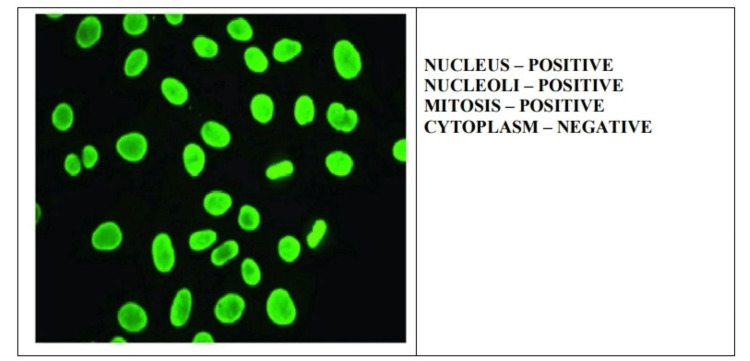
Homogenous antinuclear antibody pattern on indirect immunofluorescence.

**Figure 2:**
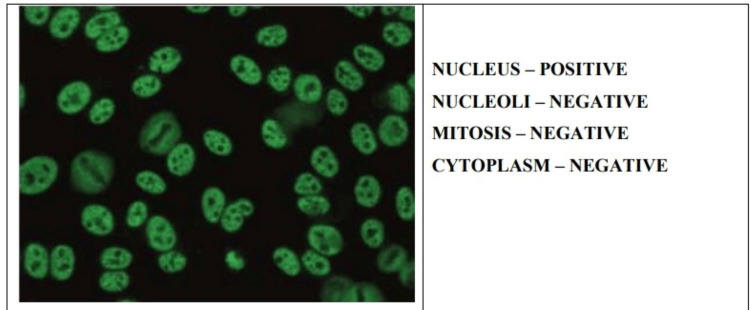
Speckled antinuclear antibody pattern on indirect immunofluorescence.

**Figure 3:**
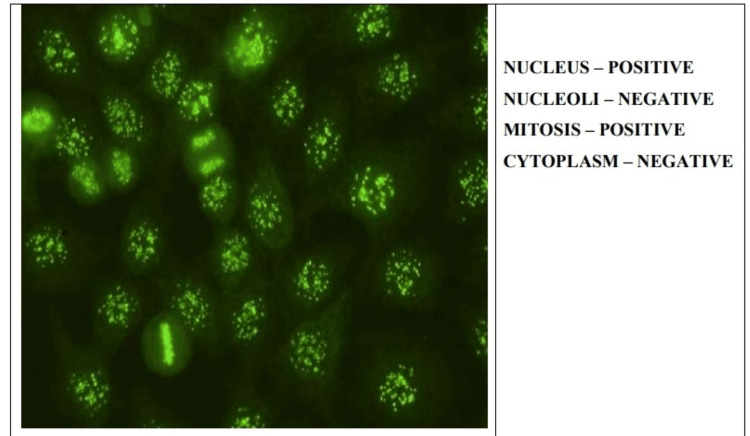
Centromere antinuclear antibody pattern on indirect immunofluorescence.

**Figure 4:**
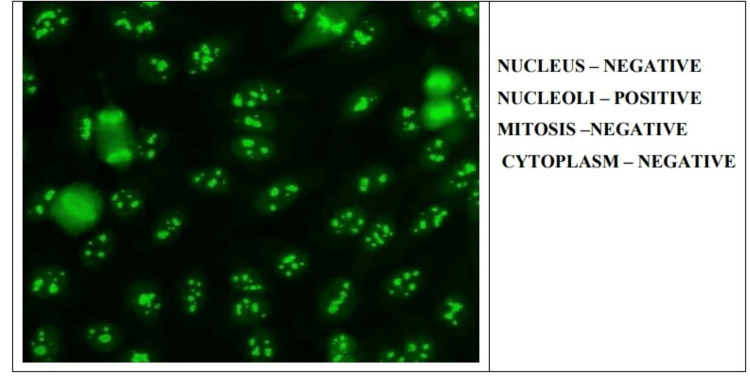
Nucleolar antinuclear antibody pattern on indirect immunofluorescence.

In this study, we mainly focused on the samples that gave fluorescence positive for either of the four patterns, i.e., homogenous, speckled, centromere, and nucleolar (Figures 1-4).

LIA for identification of specific autoantibody

**Figure 5:**
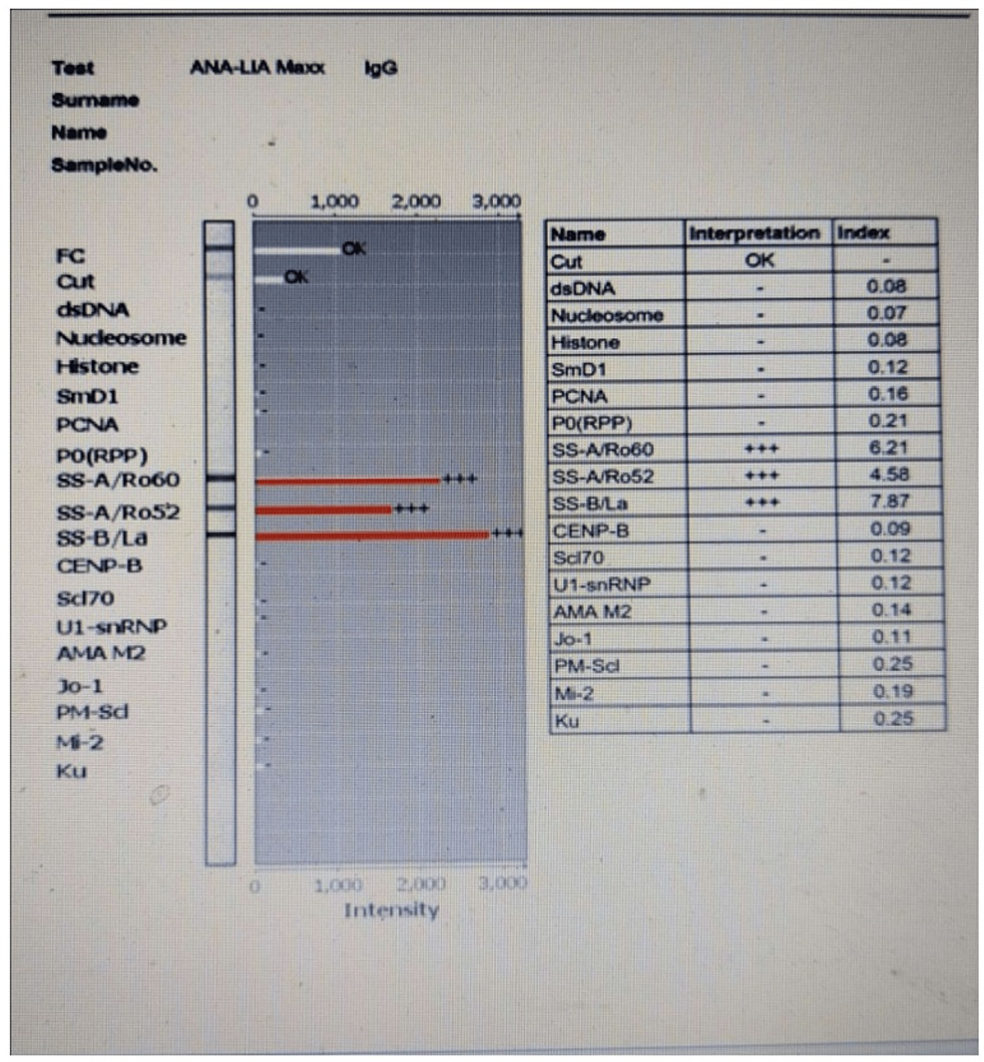
Positive antigens on line immunoassay.

LIA was performed on the Ozoblot machine provided by Medsource Ozone Biomedicals, Faridabad, Haryana. The sample used on LIA was serum with anticoagulants, citrate, or ethylenediaminetetraacetic (EDTA). Special precautions were taken about the use of samples on LIA. Highly lipemic, hemolyzed, and icteric samples were avoided. Before use, all reagents were brought to room temperature. Wash buffers, diluents, and samples were prepared as per the manufacturer’s protocol. Test strips were precoated with 17 antigens. These were dsDNA, Nucleosome, Histones, SmD1, PCNA, PO(RPP), SS-A/Ro60, SS-A/Ro52, SS-B/La, CENP-B, Scl70, U1-snRNP, AMA M2, Jo-1, PM-Scl, Mi-2, and Ku. The assay was performed as per the instruction manual and following the manufacturer’s protocol. The appearance of the band on the strip indicates the presence of autoantibodies against the respective antigen. The intensity of the band was evaluated by HumaScan (Human Diagnostics, Wiesbaden, Germany). The results were read as follows: negative, if no band was recognized or if the intensity of the band was less in comparison to the cutoff control; equivocal, if the intensity of the band and cutoff control do not differ significantly; and positive, if the intensity of the band was stronger than the cutoff control (Figure 5). The pattern obtained on IIF was correlated with the findings on LIA. The results obtained were correlated with the clinical diagnosis of the patient.

Statistical analysis

The results were presented in frequencies, percentages, and mean ± standard deviation (SD). The Chi-square test was used to assess the association. All analyses were carried out using SPSS for Windows, Version 16.0 (SPSS Inc., Chicago, IL).

## Results

A total of 98 patients with suspected ADs were included in the study. The mean age of the patients was 37.19 ± 16.25 years. Most of the patients were <30 years of age, with the majority of them being females (Table 1).

**Table 1:** Age- and gender-wise distribution of patients included in the study.

Age in years	Number of patients (*n *= 98)	Percentage (%)
<30	37	37.8
30-40	26	26.5
41-50	15	15.3
>50	20	20.4
Mean ± SD	37.19 ± 16.25	
Gender		
Male	19	19.4
Female	79	80.6

Of 98 suspected cases of AD, 82 (83.7%) had positive ANA screening by IIF and 54 (55.1%) had positive ANA profile by LIA (Table 2). Out of 82 positives for ANA screening, 50 (61%) were positive for LIA as well (true positives) and 32 (39%) were negative for LIA. Of the 54 patients with positive ANA profiles, 4 (7.4%) were negative for IIF and gave a positive result with LIA. Of the 98 cases, 12 (12.24%) gave a negative result for both tests, leading to the conclusion that they did not have AD (Table 2).

**Table 2:** Distribution of patients according to ANA screening and ANA profile results. ANA, antinuclear antibody

ANA screening and profile results	Number of samples (*n* = 98)	Percentage (%)
ANA screening		
Positive	82	83.7
Negative	16	16.3
ANA profile		
Positive	54	55.1
Negative	44	44.9

The sensitivity and specificity of the ANA profiling by LIA in comparison to IIF were found to be 61% and 75%, respectively, which makes it a better confirmatory test rather than a screening test. The positive predictive value and negative predictive value of ANA profiling were found to be 92.6% and 27.3%, respectively (Table 3).

**Table 3:** Comparison of ANA profiling and ANA screening in correctly designating a sample as positive or negative. ANA, antinuclear antibody; CI, confidence interval; PPV, positive predictive value; NPV, negative predictive value

ANA profile	ANA screening				Total	
	Positive		Negative			
	n	%	n	%	n	%
Positive	50 (true positive)	51.0	4 (false positive)	4.1	54	55.1
Negative	32 (false negative)	32.7	12 (true negative)	12.2	44	44.9
Total	82	83.7	16	16.3	98	100.0
Predictive values, % (95%CI)						
Sensitivity	61.0 (50.4-71.5)					
Specificity	75.0 (53.8-96.2)					
PPV	92.6 (85.6-99.6)					
NPV	27.3 (14.1-40.4)					

Among the patterns observed on IIF, Speckled was the most common ANA screening pattern (59.8%), followed by nucleolar (13.4%), homogenous (8.5%), and centromere (2.4%). Other patterns (15.9%) observed included cytoplasmic patterns and patterns associated with mitotic cells.

Table 4 shows the association of the ANA profile antigens with the ANA screening pattern. The clustered antigens most commonly associated with the speckled pattern were SS-A/Ro60 (34.7%), followed by U1-snRNP (32.7%), and SS-B/La (30.6%). The clustered antigens most commonly associated with the nucleolar pattern were SS-B/La (27%) followed by PCNA (18%), CENP-B (18%), U1-snRNP (18%), and AMA M2 (18%), whereas the clustered antigens most commonly associated with a homogenous pattern were U1-snRNP (85.7%), dsDNA (71.4%), Nucleosome (71.4%), Histone (71.4%), SmD1(71.4%), SS-B/La (71.4%), and Mi-2 (71.4%). The most common autoantibody found in total ANA profile-positive patients were anti-U1-snRNP (*n *= 25 ), anti-SS-B/La (*n *= 24), and anti-SS-A/Ro60 (*n *= 23). All three were mostly associated with a speckled pattern.

**Table 4:** Shows the association of ANA profile antigens with ANA screening patterns. ^*^Multiple antigens were detected on LIA, in a single ANA screening pattern found on IIF. ANA, antinuclear antibody; dsDNA, double-stranded deoxyribonucleic acid

ANA profile antigen*	ANA screening pattern with the number of patients, n (%)				
	Centromere (n = 2)	Homogenous (n = 7)	Nucleolar (n = 11)	Speckled (n = 49)	Others (n = 13)
dsDNA	-	5 (71.4)	-	-	-
Nucleosome	-	5 (71.4)	-	-	-
Histones	-	5 (71.4)	-	-	-
SmD1	-	5 (71.4)	-	7 (14.3)	1 (7.7)
PCNA	-	2 (28.5)	2 (18)	2 (4)	1 (7.7)
P0	-	1 (14.3)	-	3 (6.1)	1 (7.7)
SS-A/Ro60	1 (50)	4 (57.1)	-	17 (34.7)	1 (7.7)
SS-A/Ro52	-	1 (14.3)	-	10 (20.4)	1 (7.7)
SS-B/La	-	5 (71.4)	3 (27)	15 (30.6)	1 (7.7)
CENP-B	2 (100)	1 (14.3)	2 (18)	-	3 (23)
Scl-70	-	4 (57.1)	-	2 (4)	1 (7.7)
U1-snRNP	-	6 (85.7)	2 (18)	16 (32.7)	1 (7.7)
AMA M2	-	-	2 (18)	1 (2)	2 (15.4)
PM-Scl	-	-		2 (4)	1 (7.7)
Mi-2	-	5 (71.4)	1 (9)	-	
Ku	-	4 (57.1)	1 (9)	2 (4)	1 (7.7)

Table 5 shows the association of the ANA screening pattern with the clinical diagnosis. The most common disorders that were found to be associated with the speckled pattern were SLE (26.5%), systemic sclerosis (8.1%), and Sjogren's syndrome (4%). In the homogenous pattern, the most common disease found associated was SLE (42.9%) followed by MCTD (28.6%). The nucleolar pattern was equally associated with Sjogren's syndrome, primary biliary cirrhosis (PBC), and MCTD. The centromere pattern was invariably associated with systemic sclerosis.

**Table 5:** Association of the ANA screening pattern with the clinical diagnosis. ANA, antinuclear antibody; MCTD, mixed connective tissue disorder; PBC, primary biliary cirrhosis; SLE, systemic lupus erythematosus

ANA screening patterns and the number of patients in parenthesis (*n *= 82)	Diagnosis													
	MCTD		PBC		SLE		Rheumatoid arthritis		Sjogren's syndrome		Systemic sclerosis		Others	
	n	%	n	%	n	%	n	%	n	%	n	%	n	%
Centromere (2)	0	0	0	0	0	0	0	0	0	0	2	100	0	0
Homogenous (7)	2	28.6	0	0	3	42.9	0	0	0	0	1	14.3	1	14.3
Nucleolar (11)	1	9	1	9	0	0	0	0	1	9	0	0	8	72
Speckled (49)	2	4	0	0	13	26.5	0	0	2	4	4	8.1	28	57.1
Others (13)	0	0	1	7.7	2	15.4	0	0	0	0	1	7.7	9	69.2

Table 6 shows the association of ANA profile antigen with the clinical diagnosis. The most common autoantibody found in total ANA profile-positive patients were anti-U1-snRNP (*n *= 25 ), anti-SS-B/La (*n *= 24), and anti-SS-A/Ro60 (*n *= 23), and all three were found to be associated with SLE. Mi-2 was found to be more commonly associated with MCTD, and Scl-70 was found to be equally associated with systemic sclerosis as SLE. CENP-B was found to be most commonly associated with systemic sclerosis. Most of the antigens detected in patients on LIA were found to be commonly associated with SLE. P0 and PM-Scl were invariably seen to be associated with SLE.

**Table 6:** Association of the ANA profile antigen with the clinical diagnosis. ^*^Each diagnosed patient was found to have more than one antigen positive on LIA. ANA, antinuclear antibody; MCTD, mixed connective tissue disorder; PBC, primary biliary cirrhosis; SLE, systemic lupus erythematosus; LIA, line immunoassay

ANA profile antigen with the number of samples with these antigens in parenthesis	Diagnosis*													
	MCTD		PBC		SLE		Rheumatoid arthritis		Sjogren's syndrome		Systemic sclerosis		Others	
	n	%	n	%	n	%	n	%	n	%	n	%	n	%
dsDNA (*n *= 5)	2	40	0	0	3	60	0	0	0	0	0	0	0	0
Nucleosome (*n *= 5)	2	40	0	0	3	60	0	0	0	0	0	0	0	0
Histones (*n *= 5)	2	40	0	0	3	60	0	0	0	0	0	0	0	0
SmD1 (*n *= 13)	2	15.4	0	0	10	77	0	0	0	0	1	7.7	0	0
PCNA (*n *= 7)	2	28.6	0	0	4	57.1	0	0	0	0	0	0	1	14.3
P0 (*n *= 5)	0		0	0	5	100	0	0	0	0	0	0	0	0
SS-A/ Ro60 (*n *= 23)	2	8.7	0	0	13	56.5	0	0	0	0	5	21.7	3	13
SS-A/ Ro52 (*n *= 12)	1	8.3	0	0	7	58.3	0	0	1	8.3	2	16.6	1	8.3
SS-B/La (*n *= 24)	1	4.1	0	0	13	54.2	0	0	3	12.5	2	8.3	5	20.8
CENP-B (*n *= 8)	0	0	1	12.5	0	0	0	0	0	0	4	50	3	37.5
Scl-70 (*n *= 7)	1	14.3	0	0	3	42.8	0	0	0	0	3	42.8	0	0
U1-snRNP (*n *= 25)	5	20	0	0	14	56	0	0	0	0	2	8	4	16
AMA M2 (*n *= 5)	1	20	2	40	0	0	0	0	1	20	0	0	1	20
PM-Scl (*n *= 3)	0	0	0	0	3	100	0	0	0	0	0	0	0	0
Mi-2 (*n *= 6)	3	50	0	0	2	33.3	0	0	0	0	0	0	1	16.7
Ku (*n *= 8)	0	0	0	0	3	37.5	0	0	0	0	0	0	5	62.5

## Discussion

ADs are each characterized by distinct sets of autoantigens and antibodies, which bestow these diseases with specific immune profiles or fingerprints. These immune fingerprints have cleared the diagnostic dilemma of these diseases to some extent.

In this study, the majority of patients were aged <30 years (37.8%), and this was in agreement with studies by Angel et al. and Greidinger [9,10]. Female predominance in the ratio of 4:1 was seen, which was in concordance with the study by Gunnarsson et al. [11]. Researchers have also suggested that the X chromosome and X inactivation are related to ADs. Due to the typical presence of two X chromosomes in females, they are more likely than males to develop autoimmune disorders [12]. Women aged between 20 and 50 years are most likely to develop ADs as a result of hormonal shifts brought on by puberty, pregnancy, and menopause. Because of the unfavorable interaction between innate and adaptive immunity and the release of pro- and anti-inflammatory cytokines, these endocrinological changes, which take place during various phases of a woman's life, make her immune system more susceptible to ADs [13].

ANA screening by HEp-2 IIF was positive in 82/98 (83.7%) patients. Of these ANA-IIF positives, 50/98 (51%) patients were found positive by the ANA profile as well. The remaining 32 patients who were IIF-positive gave a negative result of ANA profile on LIA. The reason may be that LIA misses incorporating some rare antigens on their strips, which may have led to this discrepancy between the results of ANA screening and ANA profile. Four patients who were IIF-negative gave positive results on the ANA profile by LIA, and negative fluorescence may be the result of a low concentration of autoantigens or its destruction during the preparation process. This result was similar to the study by Petchiappan et al. and Latha et al. [14,15].

In our study, the speckled pattern (49/82, 59.8%) was identified as the most common result in ANA screening by HEp-2 IIF, followed by the nucleolar pattern (11/82, 13.4%). A similar pattern was also found in the study by Petchiappan et al. [14], which shows speckled as the most common pattern followed by nucleolar. In a study by Jeon et al. [16] speckled pattern was the most common pattern followed by the cytoplasmic pattern, which is not in concurrence with our study.

SLE was the most common among the diagnosed ADs, similar to the study by Angel et al. followed by MCTD and systemic sclerosis [9]. Similar findings were observed in the study conducted by Abdelbaky et al., where SLE was predominant over other connective tissue disorders [17].

In our study, out of 17 antigens of the ANA profile, U1-snRNP, dsDNA, Nucleosome, Histone, SmD1, SS-B/La, and Mi-2 were found among all patients whose ANA screening pattern was homogenous. Therefore, in the homogenous pattern, mostly clustered antigens were U1-snRNP, dsDNA, Nucleosome, Histone, etc. In speckled, mostly clustered antigens were SS-A/Ro60, followed by U1-snRNP and SS-B/La, while in nucleolar were SS-B/La followed by PCNA, CENP-B, U1-snRNP, and AMA M2. The centromere pattern was mostly associated with CENP-B. A similar result was found in a study done by Sebastian et al. [18]. The speckled and homogenous patterns were most commonly found to be associated with SLE, and the nucleolar pattern was not found associated with any AD specifically, while the centromere pattern was found associated with systemic sclerosis.

In a study by Jeon et al., the most prevalent autoantibodies in the ANA profile by LIA-positive samples were anti-Ro52 (44.3%), anti-SS-A (42.4%), and anti-SS-B (17%) [16] but in our study, the most common autoantibody found in total ANA profile positive samples were anti-U1-snRNP 25/54 (46.2%) followed by anti-SS-B/La 24/54 (44.4%) and anti-SS-A/Ro60 (42.6%).

In a study by Jeon et al., anti-SS-A (47.4%), anti-Ro52 (42.1%), anti-dsDNA (39.5%), anti-nucleosome (28.9%), anti-ribosomal P (28.9%), anti-nRNP/Sm (26.3%), anti-histone (26.3%), and anti-SS-B (26.3%) were identified in SLE patients [16], whereas in our study, P0 (100%), PM-Scl (100%), anti-U1-snRNP (56%), anti-SS-A/Ro60 (56.5%), anti-SS-B/La (54.2%), anti-SmD1 (77%), and anti-SS-A/Ro52 (58.3%) were associated with SLE patients.

In the same study by Jeon et.al [16], anti-SS-A (78.8%), anti-Ro52 (66.7%), and anti-SS-B (42.4%) were associated with Sjogren's syndrome, whereas in our study, SS-B/La, SS-A/Ro52, and AMA M2 were associated with Sjogren's syndrome.

In our study, SS-A/Ro60, CENP-B, and Scl-70 in decreasing order were the most common antigens associated with patients of systemic sclerosis, and U1-snRNP, Mi-2, dsDNA, nucleosome, histones, SmD1, PCNA, and SS-A/Ro60 were associated antigens with MCTD in decreasing order.

## Conclusions

The most prevalent autoimmune condition found in our study samples was SLE, with a speckled pattern on IIF and anti-U1-snRNP as the most prevalent ANA found by LIA. The most cost-effective way to screen patients suspected of having ADs in routine clinical practice is IIF. The fluorescent pattern in IIF predicts the presence of specific antibodies; however, due to its high sensitivity rate, it often generates false-positive reports. LIA being a costly test is out of reach for many people in a developing country like India and should be reserved for IIF-positive but dubious cases whose signs/symptoms are vague and do not match the disease dictated by IIF.
